# Three cases of ARDS: An emerging complication of *Plasmodium vivax* malaria

**DOI:** 10.4103/0970-2113.68323

**Published:** 2010

**Authors:** Supriya Sarkar, Kaushik Saha, Chandra Sekhar Das

**Affiliations:** *Department of Chest Medicine, N.R.S. Medical College, Kolkata-700 014, India*

**Keywords:** Acute respiratory distress syndrome, malaria, mechanical ventilation, positive end expiratory pressure, vivax

## Abstract

*Plasmodium (P.) vivax* malaria is rarely associated with severe complications like acute respiratory distress syndrome (ARDS). We report three cases of ARDS, which occurred as a complication of vivax malaria, from the city of Kolkata. A middle aged man who developed ARDS along with hepatic and renal dysfunction on the day 7 after completion of antimalarial treatment; a 36-year-old man who developed ARDS on the day 5 after completion of antimalarial treatment and a 15-year-old boy who developed ARDS on day 2, before starting anti-malarial drug. In all cases, vivax malaria was diagnosed by peripheral blood film (PBF) examination. Associated falciparum infection was excluded by repeated PBF examination, and by negative *P. falciparum* malaria antigen tests. In all cases, ARDS was diagnosed by the presence of hypoxia with PaO_2_ / FiO_2_ ratio < 200 and bilateral pulmonary infiltration, and by excluding cardiac disease by echocardiography. All cases typically had dramatic onset of ARDS, and required immediate (within hour of onset of dyspnea) institution of mechanical ventilation with high positive end expiratory pressure. All three cases recovered completely, and early ventilator support was life-saving.

## INTRODUCTION

Malaria is a mosquito-borne parasitic disease mainly caused by *P. vivax* and *P. falciparum*, in India. Severe malaria, a complicated form characterized by serious organ failures or abnormalities in the patient’s blood or metabolism, usually occurs in *P. falciparum* malaria. The manifestations of severe malaria include: cerebral malaria, severe anemia, hemoglobinuria, pulmonary edema or ARDS, abnormalities in blood coagulation and thrombocytopenia, cardiovascular collapse and shock, acute kidney failure, hyperparasitemia, metabolic acidosis and hypoglycemia.

In contrast to falciparum malaria, vivax malaria is rarely associated with serious complication. It was thought that *P. vivax* may cause severe complications only where the possibility of mixed infections exists.[1] But scattered cases of *P. vivax* causing severe malaria have been reported in the last 30 years. ARDS as a complication of *P. vivax* infection was reported in a traveler, with low immunity against malaria, returning from Venezuela[2] and from Gujarat, India.[3] *Plasmodium vivax*-associated ARDS is a clinically recognizable condition whose underlying pathophysiology is likely to reflect processes that are independent of parasite sequestration in the pulmonary microvasculature.[3]

In this article, we report three cases of sudden onset of ARDS from Kolkata as a complication of vivax malaria. All cases needed ventilator support with high PEEP with one hour of onset of dyspnea, and recovered completely.

## CASE REPORTS

### Case 1

**Figure 1:**
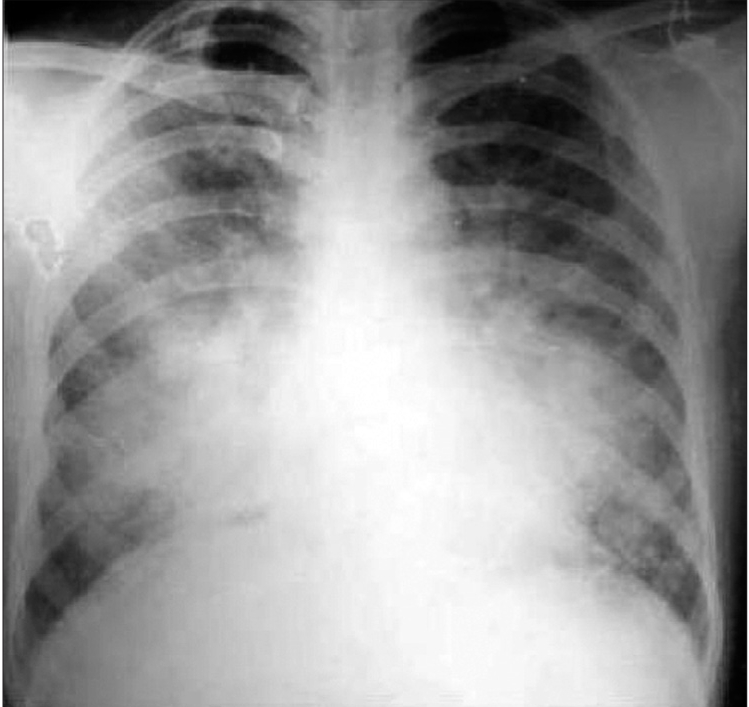
Chest X-ray PA view of Case 1, showing bilateral para-Hilar alveolar filling pattern without cardiomegaly

A 42-year-old man from North Kolkata presented with high grade intermittent fever with chills and rigor for seven days; vomiting and headache for four days. His PBF showed both ring and trophozoite form of *P. vivax*, and he received full course of antimalarial treatment with chloroquine. Associate *falciparum* infection was excluded by repeated PBF examination and by negative *falciparum* antigen test. On day 7, he suddenly developed progressive respiratory distress. Examination revealed that patient was dyspneic with BP - 100/60 mm of Hg; pulse - 110/min; respiratory rate (RR) - 32/min; and fine crepitation in both lung bases. His peripheral blood examination and routine biochemistry revealed hemoglobin (Hb) - 11.5gm/dl, WBC - 10,800/cmm, N_72,_L_25,_M_1,_E_2_, ESR - 65mm in 1^st^ hour, platelet count - 190,000/cmm, urea - 42.3mg/dl, creatinine - 1.39mg/dl, bilirubin – 1.7mg/dl, SGOT - 81.6 U/L, SGPT - 98.5U/L, alkaline phosphatase - 317.2U/L, sodium - 146.8mEq/L and potassium - 3.25mEq/L. Blood culture was negative. His initial arterial blood gas analysis (ABG) with fraction of inspiratory O_2_ (FiO_2_) 0.4 showed pH - 7.411, PaCO_2_ - 41mm of Hg, HCO_3_^-^ - 26.1mmol/L, and PaO_2_ - 63mm of Hg and SaO_2_- 9 2%. Calculated PaO_2_ / FiO_2_ ratio was 157.5. Portable X-ray chest AP view showed bilateral diffuse opacities [Figure 1]. His O_2_ saturation fell rapidly despite O_2_ treatment, and patient was put on mechanical ventilation ACMV mode with tidal volume (Tv) 350ml, RR-30/min, FiO_2_-1, PEEP - 10cm of H_2_O. Echocardiography was normal. Patient gradually improved, and we were able to reduce FiO2 to 0.6 within 24 hours. We started weaning process from day 10, and that was completed by another three days.

### Case 2

**Figure 2:**
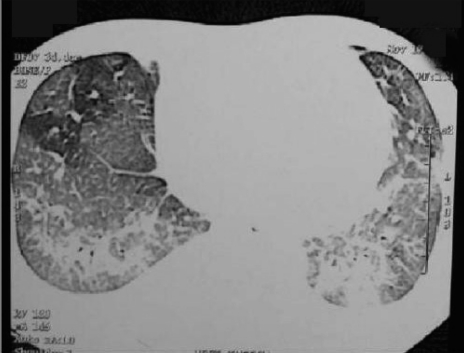
CT scan of chest of Case 2, showing bilateral basal ground glass opacities with interlobular septal thickening

A 36-year-old man from central Kolkata was diagnosed with malaria, with both ring and trophozoite forms of *P. vivax* present in his PBF. Repeated PBF examination for *P. falciparum* infection and *falciparum* antigen test were negative. He was treated with chloroquine. He developed progressive shortness of breath and one small bout of hemoptysis on day 4. Examination revealed BP - 130/80, pulse - 110/min, RR - 32/min, and bilateral diffuse crepitations in all areas of thorax. His X-ray showed bilateral diffuse reticulo-nodular opacities, and CT-thorax revealed bilateral ground glass and nodular opacities [Figure 2]. His initial ABG with FiO _2_ 0.44 showed pH - 7.43, PaCO_2_ - 29.5mm of Hg, HCO_3_ ^-^ - 20.5mmol/L, and PaO_2_ - 64mm of Hg and SaO_2_ - 90%. Patient deteriorated rapidly with ABG after half an hour showed pH - 7.49, PaCO _2_ - 28mm of Hg, HCO_3_ ^-^ - 15.5mmol/L, and PaO_2_ - 44mm of Hg and SaO_2_ - 68% with FiO2 - 0.44, and PaO2 / FiO_2_ ratio was 100. He was put on mechanical ventilator with ACMV mode with PEEP 12 cm of H _2_ O. His echocardiography was normal. Patient improved gradually and he was extubated on the day 10.

### Case 3

A 15- year-old boy from central Kolkata, with intermittent fever accompanied by chills and rigor for two days, presented with progressive respiratory distress. On examination, he was dyspneic with BP - 110/60mm of Hg, pulse - 115/min, RR - 48/min, and crepitation of both lungs, mainly lower and mid areas. Investigations revealed Hb - 11.1gm/dl, WBC - 6700/cmm, N_72_L_22_M_2_E4, platelet - 160,000/cmm, bilirubin - 0.6mg/dl, SGPT - 38U/L, creatinine - 0.8mg/dl, Na^+^ - 134mEq/L and K^+^ - 3.5mEq/L. His PBF showed trophozoites of P. vivax, and malaria antigen test was negative for *falciparum*. His X-ray chest PA view showed bilateral diffuse extensive opacities. His ABG with FiO_2_ 0.44 showed pH - 7.432, PaCO _2_ - 27.8mm of Hg, HCO3^-^ - 18.5mmol/L, and PaO_2_ - 52mm of Hg, SaO_2_ - 88% and PaO_2_ / FiO _2_ ratio was 118. He was put on mechanical ventilator with ACMV mode with Tv - 350ml, PEEP - 8cm of H_2_O, RR - 35/min, FiO_2_– 1. FiO_2_ was reduced to 0.6 on the next day. Echocardiography was normal. Weaning process was completed by day 7. Recovery was complete with follow up X-ray after one month was normal.

## DISCUSSION

Manifestations of malaria vary from asymptomatic infection to acute febrile syndrome, severe malaria, and lethal cases. Clinical evolution depends on factors of the parasite, the host, and on social and geographic characteristics.[4] The greater severity and frequency of severe malaria by *P. falciparum* (malignant malaria) could be explained by synergy of immune and physical responses. The essential pathologic feature of severe malaria is sequestration of erythrocytes, which contain mature forms of the parasite in the deep vascular beds of vital organs and rosette formation, thus producing organ dysfunction. People living in endemic areas seem to develop some immunity to the parasites, which would partially explain the low frequency of these complications in endemic areas.

Anstey *et al* raised the hypothesis that the widespread use of chloroquine and doxycycline in malaria-endemic areas could be attenuating or diminishing the number of severe cases because of the anti-inflammatory properties of these drugs.[5] It is known that non-immune subjects can present severe clinical manifestations of malarial infection.[2]

*P. vivax* (benign tertian malaria) may no longer be a paradigm for uncomplicated malaria. There are scattered reports of shock with ARDS,[1] pulmonary edema,[6] ARDS[7] and bronchiolitis obliterans[8] in benign tertian malaria. Pulmonary involvement in malaria may be asymptomatic or with minimum symptoms such as cough which may be easily overlooked. Compromised pulmonary function (small airway obstruction, gas exchange alterations, and increased pulmonary phagocytic activity) was demonstrated in clinically uncomplicated cases of both *falciparum* and vivax malaria.[5] Clinical data from patients strongly indicate that *P. vivax* can cause both sequestration-related and nonsequestration-related complications of severe malaria as a result of accumulation of pulmonary monocytes and following intravascular inflammatory response.[5] The possible role of an inflammatory mechanism in pulmonary damage by vivax malaria suggests a potential benefit for the use of corticosteroid therapy. However, there is no evidence that supports this therapeutic approach. The use of corticosteroids was reported in only one case diagnosed as bronchiolitis obliterans organizing pneumonia (BOOP), secondary to malaria.[8] Severe pulmonary complications of vivax malaria usually appear from six hours to eight days after the initiation of anti-malarial treatment and they could correspond to an exacerbation of the post-treatment inflammatory response.[5] However, severe pulmonary symptoms may occur before the initiation of anti-malarial treatment.[9]

Treatment of ARDS usually requires institution of invasive mechanical ventilation with high PEEP. However, in a systematic review of the literature for the use of noninvasive ventilation (NIV) in cases of acute lung injury (ALI) / ARDS related to P. vivax, Agarwal *et al*, found that the use of NIV in vivax malaria related ALI / ARDS is associated with a good outcome.[10]

In most reported cases of ARDS due to vivax malaria, the diagnosis was made by PBF examination without molecular diagnostic confirmation. Thus co-infection with *P. falciparum* could not be ruled out, and most of the reported cases were treated for both *P. falciparum* and *P. vivax*. However, Luxemburger *et al*,[11] observed that severe malaria is 4.2 times less common in patients with mixed *P*. falciparum and *P*. vivax infections than in those with *P. falciparum* alone. Although detection of *P. vivax* in PBF is the standard, all patients should be subjected to a thorough diagnostic evaluation to rule out the possibility of mixed infection; which should include repeated and meticulous examination of PBF examination, a rapid diagnostic test for malaria based on detecting specific *Plasmodium* lactate dehydrogenase (LDH) antigen by using monoclonal antibody directed against isoforms of the enzyme, and polymerase chain reaction (PCR).

We used one-step malaria P.f/P.v rapid test[12] with a membrane strip coated with monoclonal antibodies (one for LDH of *P. falciparum* and one for LDH of Plasmodium species) in separate lines. A pink/purple visible line appears at the regions on the membrane indicates positive test. Appearance of another line (Control) validates the procedure. The test can discriminate between *P*. falciparum and *P. vivax* malaria. The test has 98.2% sensitivity and 99.5% specificity for detecting *P. falciparum* infection.

We are reporting two middle aged men and one adolescent boy suffering from vivax malaria complicated with ARDS. The first case was associated with impairment of renal and liver function. In two cases ARDS developed after antimalarial treatment, and in the third case, ARDS developed before starting chloroquine. In all cases the diagnosis of vivax malaria was made by examination of PBF, and *falciparum* co-infection was ruled out by repeated and meticulous examination of PBF and by negative falciparum antigen tests. The diagnosis of ARDS was confirmed by excluding cardiac disease by echocardiography. In all cases, characteristically ARDS developed dramatically necessitating intubation within an hour of onset of respiratory distress. All of them were treated for *P. vivax* with chloroquine. Recovery was quick and complete in all three cases. Early institution of mechanical ventilation with high PEEP was proved to be life-saving in all of them.
